# Carbon dots-based drug delivery for bone regeneration

**DOI:** 10.3389/fbioe.2025.1613901

**Published:** 2025-05-29

**Authors:** Christy Liu, Yingzi Li, Xiaohua Liu

**Affiliations:** ^1^ School of Medicine and College of Engineering, Case Western Reserve University, Cleveland, OH, United States; ^2^ Department of Chemical and Biomedical Engineering, University of Missouri, Columbia, MO, United States; ^3^ NextGen Precision Health, University of Missouri, Columbia, MO, United States

**Keywords:** carbon dots, drug delivery, bone, tissue engineering, nanomaterials

## Abstract

Carbon dots (CDs) are a class of nanobiomaterials with significant potential in bone regeneration. Their excellent biocompatibility, tunable fluorescence, high stability, low toxicity, and abundant functional groups make CDs promising candidates for efficient drug delivery and bone tissue regeneration. CDs contribute to targeted drug release, enhance osteogenic differentiation, and interact with cellular components to facilitate bone formation. Recent research highlights the roles of CDs in scaffold-based approaches, offering controlled drug delivery and real-time bioimaging capabilities. This review provides a comprehensive overview of CDs in bone regeneration, with a focus on their synthesis, functionalization, and biomedical applications. It begins by exploring CD synthesis methods, physicochemical properties, and mechanisms of action. Next, it discusses CD-based drug delivery systems and their applications in bone regeneration. Finally, the review highlights the challenges and future perspectives in optimizing CDs for enhanced therapeutic outcomes.

## 1 Introduction

Bone tissue regeneration is a vital research area within regenerative medicine, addressing critical health challenges related to bone injuries, defects, and degenerative diseases (Koons et al., 2020). While bone tissue inherently possesses the ability to repair itself to some extent, this natural healing capacity becomes insufficient in cases of severe trauma, large defects, or age-related bone loss. Such scenarios necessitate the implementation of bone tissue engineering approaches to restore the structure and function of lost or damaged bone tissues in the affected area (Liu and Ma, 2004; Liu et al., 2009; Li et al., 2022).

Successful bone tissue engineering relies on the development of suitable biomaterials and drug/growth factor delivery systems (Li et al., 2022; Ma et al., 2015; Li et al., 2021). Nano biomaterials, characterized by their nanoscale size and high surface area-to-volume ratio, can mimic the structural and functional features of natural bone tissue at the molecular level, thereby creating an environment conducive to bone regeneration (Sachar et al., 2012; Sun et al., 2013; Ma and Liu, 2017). Numerous studies have demonstrated that nano biomaterials actively regulate the cellular behaviors of bone marrow-derived stem cells (BMSCs), including adhesion, proliferation, differentiation, and biomineralization (Gong et al., 2015; Chang et al., 2018; Jin et al., 2014; Jin et al., 2004; Popat et al., 2007). Additionally, the high surface area of nano biomaterials provides significant advantages in delivering therapeutic agents such as growth factors, cytokines, and drugs to targeted sites in a controlled manner (Li et al., 2022). The integration of nano biomaterials and controlled drug delivery systems effectively addresses the complex biological and mechanical requirements of bone regeneration, offering a promising avenue for advanced therapeutic solutions (Goldberg et al., 2007).

Among the diverse range of nano biomaterials developed for tissue regeneration, carbon dots (CDs) have emerged as a highly versatile and promising class of nano biomaterials (Liu et al., 2020). CDs are nanoscale particles with the diameter typically less than 10 nm. CDs are composed primarily of carbon and offer distinct advantages over traditional nanomaterials due to their superior optical characteristics, tunable surface properties, and high biocompatibility (Lim et al., 2015). Unlike conventional nanoparticles, CDs exhibit strong fluorescence, enabling enhanced imaging and real-time monitoring in biological applications. Their small size and ease of functionalization facilitate efficient drug delivery and targeted therapy. Additionally, CDs are derived from eco-friendly precursors, ensuring lower toxicity and improved biodegradability compared to other nanomaterials.

An increasing number of publications have demonstrated that CDs-based nanomaterials promoted osteogenic differentiation and bone matrix mineralization in recent years (Shao et al., 2017; Khajuria et al., 2018; Jin et al., 2020; Ren et al., 2021; Zhang et al., 2023). CDs have also been incorporated into various scaffolds to improve mechanical properties and support bone tissue integration (Gogoi et al., 2016; Shafiei et al., 2019; Rajabnejadkeleshteri et al., 2021). Furthermore, the high surface area, tunable surface chemistry, and biocompatibility of CDs make them excellent carriers for delivering therapeutic agents to bone defects. In fact, many CDs-based deliver systems have been reported for enhanced bone formation (Rajabnejadkeleshteri et al., 2021; DuMez et al., 2021; Wan et al., 2022; Samadian et al., 2022; Juma et al., 2025). These advancements underscore the promise of CDs in developing innovative and effective solutions for bone tissue regeneration.

This review provides a comprehensive overview of the potential of CDs in bone tissue engineering, with a focus on their dual roles as nano biomaterials and drug delivery carriers for bone regeneration. It begins by briefly introducing the synthesis and properties of CDs, as well as the bone regeneration process and its requirements. It then examines the mechanisms by which CDs facilitate bone regeneration. Next, the paper delves into the discussion of CDs-based drug delivery systems. Finally, it explores the applications of CDs-based drug delivery in bone regeneration. Through this review, readers are expected to gain a deeper understanding of the transformative potential of CDs in advancing regenerative medicine and addressing the existing challenges in bone tissue engineering.

## 2 Synthesis and properties of CDs

### 2.1 Methods of CD synthesis

**FIGURE 1:**
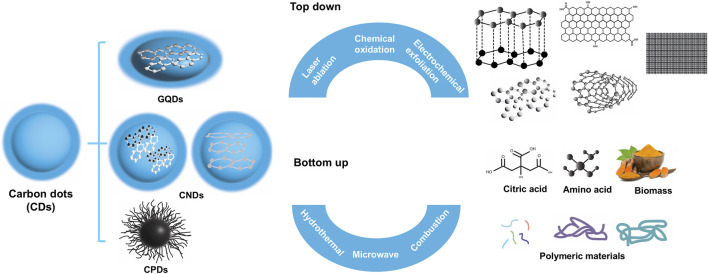
Classification of CDs and the two general approaches to synthesize CDs.

CDs can be broadly classified into graphene quantum dots (GQDs), carbon nanodots (CNDs), and carbonized polymer dots (CPDs) (Figure 1). The synthesis of CDs can be categorized into top-down approaches and bottom-up approaches, both of which offer unique advantages and constraints. The method of synthesis directly influences the size, structure, and surface chemistry of the resulting CDs, which are crucial for their applications. It is important to note that CDs fabricated using different methods are inherently distinct, making direct comparisons between fabrication techniques and the properties of CDs challenging, if not impossible.

#### 2.1.1 Top-down approaches

Top-down approaches for synthesizing CDs rely on breaking down larger carbonaceous materials into nanoscale particles through physical, chemical, or electrochemical processes. These methods are effective for producing CDs with well-defined size and morphology, leveraging the controlled disintegration of bulk carbon sources. The primary techniques within this category include laser ablation, chemical oxidation, and electrochemical exfoliation, (Lim et al., 2015; Liu M. L. et al., 2019), which are briefly discussed below.

Laser ablation is a widely utilized top-down method for CD synthesis, involving the application of high-powered laser beams to carbon-rich precursors such as graphite, graphene oxide, or carbon nanotubes (Kaczmarek et al., 2021; Kim et al., 2017). The intense energy from the laser rapidly fragments the bulk material into nanometer-sized CDs, resulting in particles with excellent optical properties and strong photoluminescence. Additionally, laser ablation allows precise control over particle size and morphology, making it advantageous for applications requiring uniform CDs. However, this method demands specialized and expensive equipment, limiting its practicality for large-scale production.

Chemical oxidation is another top-down method that involves treating bulk carbon sources (e.g., graphite powder or activated carbon) with strong oxidizing agents like nitric acid and sulfuric acid (Qiao et al., 2010). This aggressive chemical reaction breaks down carbon materials into smaller nanoparticles while introducing functional groups onto the CD surface, which enhances the water solubility and biocompatibility of CDs. Chemical oxidation is cost-effective and scalable, making it suitable for producing large quantities of CDs for industrial use. However, one of the drawbacks is the variable particle size and morphology of the CDs. In addition, the use of strong acids generates hazardous waste and raises environmental and safety concerns.

Electrochemical exfoliation is another approach for CD synthesis, in which carbon electrodes are submerged in an electrolyte solution and exposed to a controlled electrical potential (Li et al., 2019). The applied voltage induces exfoliation of the carbon material into nanoscale CDs through redox reactions, resulting in high-purity products with consistent optical properties. This technique allows for precise manipulation of synthesis parameters, including current density, potential, and electrolyte composition, enabling fine-tuning of CDs to match specific applications. However, this method requires meticulous optimization to achieve consistent results. Additionally, the high cost of ionic liquids and the time-consuming process of obtaining distilled water both hinder the large-scale preparation of materials using electrochemical exfoliation.

Overall, top-down approaches offer a versatile and effective means for synthesizing CDs with tailored properties, particularly for applications requiring high purity and controlled morphology. However, their inherent limitations, including challenges related to scalability, high energy consumption, time-intensive processes, and environmental impact, highlight the need to explore complementary strategies for optimizing CD production.

#### 2.1.2 Bottom-up approaches

Bottom-up approaches for synthesizing CDs differ from top-down methods in that they rely on assembling CDs from molecular precursors through controlled chemical reactions. The bottom-up techniques often offer better control over particle size and surface chemistry. Additionally, bottom-up approaches tend to be more environmentally friendly, as they avoid the harsh physical fragmentation processes associated with top-down synthesis. Among the most widely used bottom-up methods are hydrothermal synthesis, microwave-assisted synthesis, combustion or pyrolysis, and sonochemical synthesis, (Xia et al., 2019), which are briefly discussed below.

Hydrothermal synthesis is one of the most commonly employed bottom-up techniques due to its simplicity and environmentally friendly approach (Yang et al., 2011). This method involves dissolving organic precursors (e.g., citric acid, glucose, and amino acids) in water, followed by heating the solution in a sealed autoclave under high pressure and temperature. The elevated thermal conditions induce the carbonization of precursor molecules, leading to the formation of CDs with tunable fluorescence and surface chemistry. A key advantage of hydrothermal synthesis is its ability to produce CDs with a diverse array of functional groups, making them highly versatile for applications in bioimaging and drug delivery. However, purifying the synthesized CDs can be complex and time-consuming. Additionally, this method struggles with maintaining precise control over reaction conditions during CD synthesis.

Microwave-assisted synthesis provides a faster and more energy-efficient alternative, leveraging microwave radiation to induce localized heating and rapid carbonization of organic precursors (Jiang et al., 2018). This method enables the rapid production of CDs in minutes. Its simplicity and efficiency make microwave synthesis a promising approach for large-scale CD production. However, a major challenge associated with this technique is achieving uniform heating, which can lead to inconsistencies in CD morphology and fluorescence characteristics.

Combustion or pyrolysis is another approach for CD synthesis, involving the controlled burning or heating of organic precursors such as biomass or polymeric materials (Song et al., 2015). The high-temperature environment facilitates the rapid formation of CDs, making this method appealing for large-scale applications. It offers the advantages of simplicity and the capability to process a diverse range of precursor materials. However, CDs generated via combustion often exhibit irregular size distributions and variability in optical properties. Additionally, the inability to precisely control reaction parameters poses challenges in ensuring consistent results.

Sonochemical synthesis utilizes high-frequency ultrasonic waves to generate cavitation effects, creating localized high-temperature and high-pressure conditions that trigger chemical reactions leading to CD formation (Dehvari et al., 2019). This technique is recognized as a green chemistry approach, enabling the rapid synthesis of CDs with stable fluorescence properties. Additionally, it allows for *in situ* functionalization of CDs during their formation. However, like other methods, sonochemical synthesis of CDs often leads to low product yields and limited solid loading, which hinders scalability.

Overall, bottom-up synthesis methods offer significant advantages in environmental sustainability, tunability, and versatility for designing CDs with tailored properties. These approaches are well-suited for biomedical applications. However, challenges related to scalability and production efficiency necessitate further research and optimization.

Selecting an appropriate CD synthesis method for CD-based drug delivery in bone regeneration requires consideration of several key factors, including biocompatibility, surface functionalization, drug-loading capability, and scalability. For example, bottom-up synthesis methods are more suitable for fabricating CDs with excellent biocompatibility when using small-molecule precursors. On the other hand, top-down methods are generally more efficient for large-scale CD production. By carefully evaluating these parameters, CDs with varying functionalities can be synthesized to meet the specific demands of bone regeneration.

### 2.2 Physicochemical characteristics of CDs

CDs exhibit unique physicochemical characteristics that make them highly versatile and effective for biomedical applications. Notably, their photoluminescence, biocompatibility, antioxidant and antibacterial properties are particularly valuable for tissue regeneration. A brief summary of these key properties is provided below.

#### 2.2.1 Photoluminescence

CDs exhibit strong photoluminescence, which arises from quantum confinement effects, surface defects, the presence of conjugated π-electron systems, and crosslink enhanced emission (CEE) effect (Xia et al., 2019). CDs emit fluorescence that can be fine-tuned across a wide range of wavelengths, from visible to near-infrared, by adjusting their size, surface chemistry, or heteroatom doping (Lim et al., 2015). In addition, the excitation wavelength-dependent multicolor photoluminescence of CDs makes them well-suited for multicolor imaging of various cells and tissues (Du et al., 2015). Furthermore, embedding salt in CDs enhances their resistance to heat and UV-induced deterioration while also reducing photobleaching (Kim et al., 2014). These unique properties make CDs suitable for real-time cellular imaging and monitoring the tissue regeneration process.

#### 2.2.2 Biocompatibility

Biocompatibility is a critical attribute that determines the suitability of CDs for tissue regeneration. CDs are generally considered biocompatible, owing to their carbon-based composition and surface functionalities. CDs exhibit negligible toxicity at relative low concentrations to ensure their safe interaction with living cells (Park et al., 2020). CDs are endocytosed by several different mechanisms, such as clathrin-mediated endocytosis, caveolae-mediated endocytosis, clathrin/caveolae-independent endocytosis, and macro/micro-pinocytosis (Truskewycz et al., 2022; Joseph and Liu, 2020). Numerous factors affect endocytosis of CDs, including particles size, surface charge and functionalization, porosity, and crystallinity (Mohammadinejad et al., 2019). For example, smaller CDs are often more readily endocytosed and better penetrated into cells and tissues, while larger ones may be more easily cleared by the immune system (Liao et al., 2021). CDs with high amine passivation and positive surface charge had higher uptake and localized more strongly to lysosomes (Clermont-Paquette et al., 2024). Cell viability improved with the addition of the antioxidant activity of CDs (Caetano et al., 2024). Surface modifications, such as passivation with polyethylene glycol, enhance their biocompatibility (Peng et al., 2020). CDs were easily internalized by human BMSCs without causing significant disruption to cellular functions (Han et al., 2019). *In vivo* experiments also demonstrated that CDs possessed satisfactory biocompatibility, causing no pathological damage upon entering the body and being excreted through normal metabolic pathways (Sun et al., 2022). This property makes CDs suitable for *in vivo* bioimaging and targeted drug delivery applications.

#### 2.2.3 Antioxidant properties

Oxidative stress, resulting from an imbalance between reactive oxygen species (ROS) and antioxidants, is associated with various pathological conditions and can impede tissue regeneration. CDs effectively neutralize ROS, including superoxide anions and hydroxyl radicals, thereby preventing cellular damage and promoting tissue repair (Das et al., 2014). Their antioxidant activity is primarily attributed to unsaturated double bonds in the carbon core and surface functional groups on the shell (Wang et al., 2025). These properties enable CDs to be integrated into drug delivery systems and tissue scaffolds, offering protection against oxidative damage during tissue regeneration.

#### 2.2.4 Antibacterial properties

**FIGURE 2:**
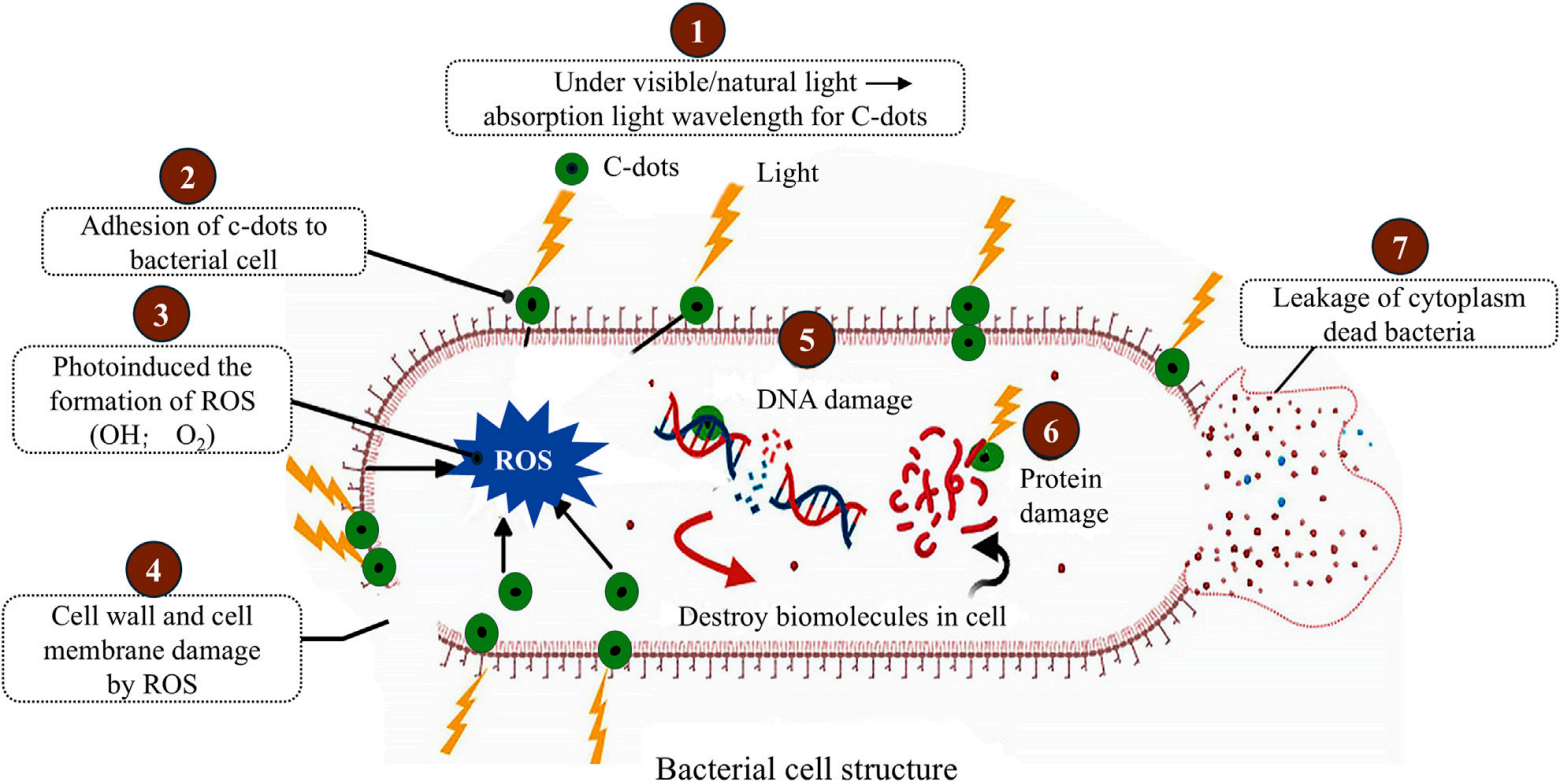
Mechanism of photoinduced antibacterial activity of CDs. Adapted from Ref. (Hussen et al., 2024), This publication is licensed under CC-BY-NC-ND 4.0. Copyright ^©^ 2024 The Authors. Published by American Chemical Society.

Some CDs have demonstrated impressive antibacterial properties, making them effective agents for combating infections and preventing bacterial growth during tissue regeneration (Dong et al., 2020). Their antimicrobial activity is a result of both physical and chemical interactions with bacteria (Figure 2) (Hussen et al., 2024). For physical disruption, CDs interact with bacterial membranes, disrupting their structural integrity and leading to cell death. For chemical interactions, some CDs, especially those doped with heteroatoms like nitrogen or sulfur, produce ROS under specific conditions, such as exposure to light (Hussen et al., 2024). These ROS damage bacterial cell walls and DNA, further enhancing antibacterial effects. The antibacterial properties of CDs allow them to be incorporated into scaffolds to prevent biofilm formation, reduce the risk of infection, and accelerate wound healing.

## 3 Bone regeneration process and requirements

### 3.1 Bone regeneration process

Bone regeneration is a meticulously coordinated biological process. This intricate process is driven by cellular activity, biochemical signaling, and progressive structural remodeling. Generally, bone healing occurs in a well-defined sequence of four distinct phases: hematoma formation, soft callus formation, hard callus formation, and remodeling (Duda et al., 2023).

**FIGURE 3:**
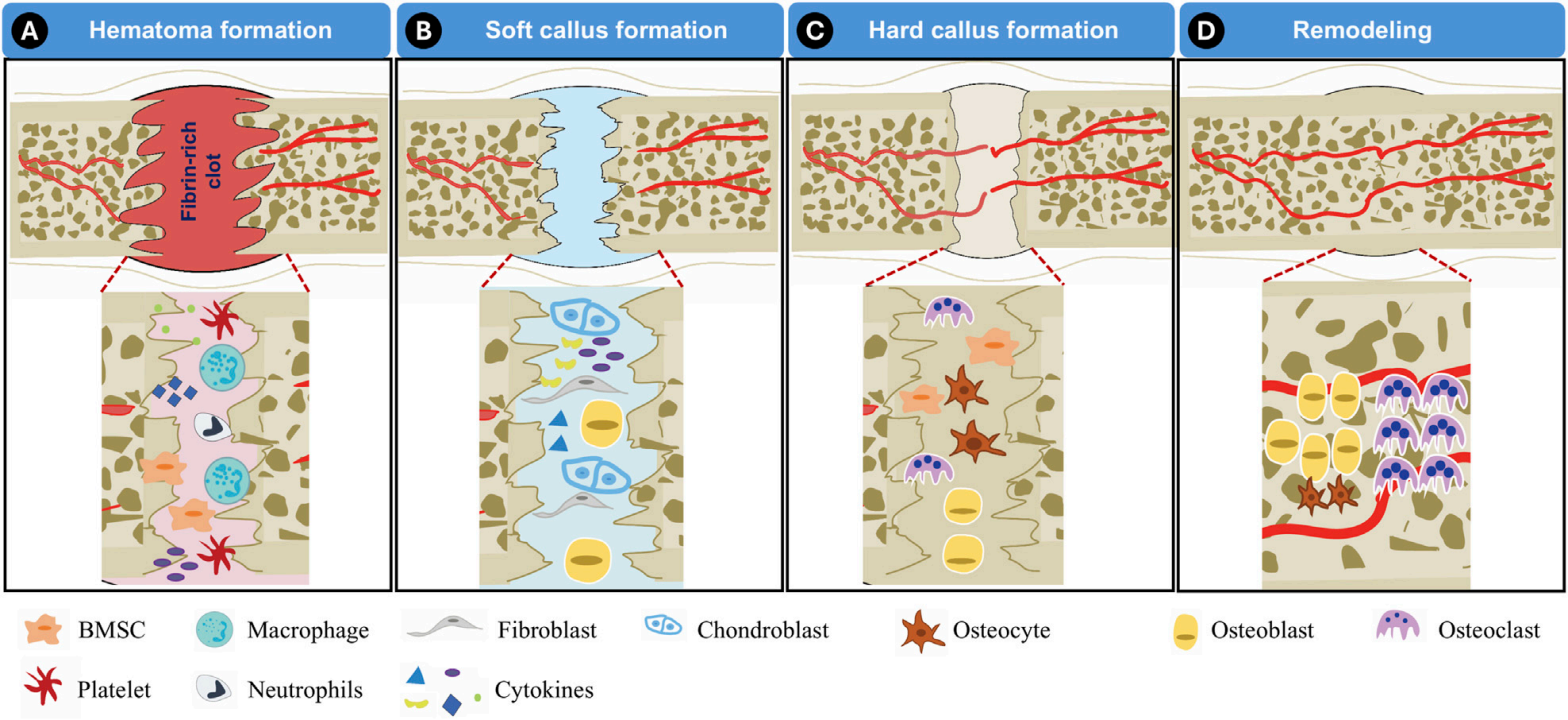
Illustration of bone regeneration process. **(A)** Hematoma formation, **(B)** Soft callus formation, **(C)** Hard callus formation, **(D)** Remodeling.

The first stage, hematoma formation, initiates immediately after an injury and serves as a crucial preparatory phase for subsequent healing. During this period, the affected area experiences bleeding, triggering the formation of a fibrin-rich clot as platelets aggregate at the injury site. This clot acts as a temporary matrix that stabilizes the damaged region (Figure 3A). Concurrently, immune cells, such as neutrophils and macrophages, migrate to the injured tissue, clearing debris while releasing cytokines such as tumor necrosis factor-alpha (TNF-α) and interleukins. These signaling molecules recruit stem/progenitor cells to the site. Additionally, angiogenesis is stimulated by vascular endothelial growth factor (VEGF), facilitating the formation of new blood vessels to ensure the delivery of oxygen and nutrients required for efficient bone healing. This hematoma formation stage typically lasts a few days (Einhorn and Gerstenfeld, 2015).

The next phase, soft callus formation, marks the transition to the reparative stage of bone healing (Figure 3B). During this period, BMSCs are actively recruited to the injury site, where they differentiate into chondroblasts. These specialized cells contribute to the development of the soft callus, a cartilage-like intermediary structure that serves to bridge the gap between fractured bone segments. The formation of this soft callus provides initial mechanical stability and lays the foundation for subsequent mineralization. This phase generally spans a few weeks, depending on the extent of the injury.

As healing progresses, the soft callus undergoes mineralization, leading to the formation of a hard callus (Figure 3C). This stage, often referred to as the bone formation phase, involves the gradual replacement of cartilaginous tissue with mineralized bone. Osteoblasts play a pivotal role in depositing calcium and phosphate, reinforcing the structural integrity of the developing bone. Depending on the severity of the injury, the hard callus formation phase takes several weeks or months to fully stabilize.

The final stage of bone regeneration is the remodeling phase, in which the newly formed bone undergoes structural refinement to restore its original mechanical properties (Figure 3D). This process is governed by the coordinated activity of osteoblasts, osteoclasts, and osteocytes. Osteoclasts resorb excess bone tissue while osteoblasts deposit new mineralized matrix, ensuring optimal strength and durability. Remodeling can extend over several months to years, particularly in cases involving extensive defects or fractures (Einhorn and Gerstenfeld, 2015).

Ultimately, bone regeneration is a dynamic interplay of cellular mechanisms, signaling pathways, and environmental cues that collectively drive the restoration of lost/damaged bone tissue. The precise coordination of these processes ensures the repair and functional recovery of skeletal structures, underscoring the complexity and efficiency of bone healing.

### 3.2 Bone regeneration requirements

As discussed above, bone regeneration is a highly intricate biological process that relies on the coordinated interplay of various components to restore damaged or lost tissue. This is particularly crucial for critical-sized bone defect regeneration, where cells, scaffolds, and bioactive molecules each play a vital role in promoting effective healing while maintaining the structural and functional integrity of the bone (Liu and Ma, 2004).

Cells serve as the fundamental driving force behind bone regeneration, enabling critical processes such as osteogenesis (bone formation), angiogenesis (blood vessel formation), and matrix remodeling. BMSCs and endothelial cells are among the most extensively studied cell types in this context (Arvidson et al., 2011). Upon injury, BMSCs migrate to the affected site, proliferate, and initiate the repair process by transforming into osteoblasts. Later, the osteoblasts produce bone matrix, differentiate into osteocytes, and eventually form new bone. Meanwhile, endothelial cells play a key role in promoting angiogenesis, ensuring sufficient oxygen and nutrient supply to the damaged region. Proper vascularization is critical for sustaining cell viability and facilitating optimal healing. The interaction between BMSCs and endothelial cells recouples osteogenesis with angiogenesis, effectively orchestrating the regeneration process and contributing to the restoration of both structural stability and functional integrity of newly formed bone tissue (Grosso et al., 2017).

Scaffolds form the backbone of bone regeneration, providing a physical framework that mimics the extracellular matrix (ECM) and supports cellular activities (Liu and Ma, 2004; Chang et al., 2017). The scaffolding structures play a crucial role in guiding new tissue formation while maintaining mechanical stability throughout the healing process. Ideally, scaffolds must be biocompatible, ensuring safe interaction with host tissues while minimizing immune responses or toxicity (Liu and Ma, 2004). Additionally, scaffolds should exhibit controlled degradation, gradually dissolving as new bone tissue replaces them to achieve seamless integration into the repaired structure. Their mechanical strength is also paramount, particularly for load-bearing bones, as they must withstand external stresses until complete bone restoration occurs. Beyond their structural role, scaffolds enhance the retention and delivery of bioactive molecules, optimizing the regenerative process (Polo-Corrales et al., 2014).

Bioactive molecules act as key regulators of cellular behavior, directing and accelerating bone regeneration (Ho-Shui-Ling et al., 2018). These include growth factors, cytokines, drugs, and mineral ions, all of which contribute to different aspects of healing. Growth factors such as bone morphogenetic proteins (BMPs) and VEGF play a vital role in stimulating bone formation and enhancing vascular integration (Kempen et al., 2009). Anti-inflammatory cytokines and therapeutic drugs help manage inflammation, ensuring a balanced healing environment while preventing excessive tissue damage (Hu et al., 2018). Additionally, mineral ions such as calcium and phosphate are essential for matrix mineralization, strengthening newly formed bone tissue (Liu et al., 2009; Jeong et al., 2019). These bioactive molecules serve as biochemical signals that guide cellular interactions, ensuring efficient and coordinated tissue regeneration.

Controlled growth factor/drug delivery systems provide an advanced mechanism to precisely administer bioactive molecules to the injury site, thereby maximizing therapeutic efficacy while minimizing systemic side effects (Ma et al., 2015; Vo et al., 2012). These systems are designed to release therapeutic agents in a targeted and sustained manner, ensuring that the effects remain localized and persist throughout the healing process. By improving the precision and efficiency of bioactive molecule delivery, controlled growth factor/drug release technologies significantly enhance bone regeneration outcomes (Grosso et al., 2017).

The successful integration of cells, scaffolds, bioactive molecules, and controlled drug delivery systems represents a comprehensive strategy for effective bone regeneration (Henkel et al., 2013). These components work synergistically to address the biological, structural, and mechanical requirements of bone repair, paving the way for innovative and advanced therapeutic solutions capable of healing the complex and challenging bone defects.

## 4 Mechanisms of CDs in promoting bone regeneration

CDs regulate bone formation through intricate molecular and cellular interactions. While the precise mechanisms by which CDs promote bone regeneration are not yet fully understood, numerous studies have highlighted their involvement in key biological processes (Shao et al., 2017; Zhang et al., 2023; Han et al., 2019; Jin et al., 2022). These include facilitating osteogenic differentiation, enhancing mineralization, and modulating signaling pathways that govern bone tissue regeneration (Shao et al., 2017; Jin et al., 2020; Han et al., 2019; Jin et al., 2022). This section explores the ways in which CDs contribute to bone repair, emphasizing their influence on cellular behavior, matrix development, and the regulatory signals that drive bone formation.

### 4.1 Control of osteogenic differentiation and mineralization

CDs control bone regeneration by influencing osteogenic differentiation and mineralization, two essential processes for effective bone regeneration (Shao et al., 2017; Han et al., 2019). The regenerative process depends on the recruitment and differentiation of BMSCs into osteoblasts, the primary bone-forming cells responsible for constructing new bone tissue. CDs contribute to this mechanism by enhancing BMSC proliferation and differentiation while simultaneously promoting matrix mineralization, ultimately leading to more functional bone formation. Research indicates that CDs upregulate key osteogenic markers such as runt-related transcription factor 2 (RUNX2), osteocalcin (OCN), and alkaline phosphatase (ALP) (Truskewycz et al., 2022). By modulating these genes, CDs enhance the regenerative potential of BMSCs, allowing for more efficient bone tissue regeneration (Shao et al., 2017; Han et al., 2019). Matrix mineralization, which involves the deposition of hydroxyapatite (HA) within the bone ECM, is another critical phase in bone regeneration. CDs facilitate this process by acting as nucleation sites for HA formation, improving the deposition of calcium and phosphate ions required for mineralization (Khajuria et al., 2018; Wu et al., 2022). Additionally, functionalized CDs carrying osteoinductive factors, such as BMPs, establish a conducive environment for controlled mineral deposition (Rajabnejadkeleshteri et al., 2021). This ability to guide precise bone mineral formation ensures that regenerated tissue maintains its structural integrity and mechanical strength, thereby reinforcing its long-term stability.

Beyond their direct contributions to osteogenesis and mineralization, CDs also exhibit antioxidant and anti-inflammatory properties to support bone regeneration (Li et al., 2025). Oxidative stress is a significant obstacle in the healing process, as it hinders osteoblast activity and compromises BMSC viability. CDs mitigate oxidative damage by neutralizing free radicals, thereby safeguarding bone-forming cells from harmful cellular stress. Moreover, their influence on inflammatory pathways plays a crucial role in modulating the immune response. For instance, resveratrol-derived CDs effectively reduced inflammatory factors such as iNOS and simultaneously increased in anti-inflammatory factors such as CD206 (Li et al., 2025). Another study indicated that Yam-CDs enhance osteoblast differentiation in an inflammatory environment by regulating the expression of histone demethylase 4B (Chen et al., 2024). By maintaining a balanced immune response, CDs prevent excessive inflammation and foster a favorable microenvironment for efficient bone tissue regeneration.

### 4.2 Signal pathways of CDs in regulating bone regeneration

CDs are involved in several key cell signaling pathways that regulate osteogenesis, angiogenesis, and immune responses (Zhang et al., 2023; Rajabnejadkeleshteri et al., 2021; Hong et al., 2025; Gao et al., 2023). These molecular mechanisms govern how bone-forming cells develop, how vascular networks integrate into new tissue, and how inflammation is modulated during the healing process. Understanding these interactions is essential for optimizing CD-based regenerative therapies and ensuring their clinical success.

One of the most influential signaling pathways involved in bone tissue formation is the Wnt/β-catenin pathway. This pathway regulates stem cell differentiation and osteogenesis, making it essential for bone repair. CDs have been shown to enhance Wnt signaling by stabilizing β-catenin in the cytoplasm and facilitating its translocation into the nucleus (Hong et al., 2025; Gao et al., 2023). This activation triggers the expression of osteogenic genes such as RUNX2, osteopontin (OPN), and collagen type I (COL1), all of which contribute to bone matrix formation and mineralization.

Another pathway involved in bone regeneration is the BMP signaling pathway, which drives osteoblast differentiation and bone formation. CDs carrying BMP-2 have demonstrated their ability to activate Smad proteins, which translocate to the nucleus and stimulate bone-specific gene expression (Zhang et al., 2023; Rajabnejadkeleshteri et al., 2021). The functionalization of CDs with BMPs ensures prolonged bioactivity at bone injury sites, enhancing the regenerative potential of these nanoparticles (Rajabnejadkeleshteri et al., 2021). Their ability to maintain steady BMP signaling contributes to improved bone matrix deposition, strengthening the integrity of newly regenerated bone tissue.

Besides the Wnt/β-catenin and BMP pathways, extracellular signal-regulated kinases (ERK)/AMP-activated protein kinase (AMPK) signaling and the protein kinase R-like ER kinase (PERK) pathways were also reported to be involved in CDs-induced osteogenic differentiation (Shao et al., 2017; Jin et al., 2020; Ren et al., 2021).

Beyond osteogenic pathways, CDs also play a key role in immune modulation, primarily by interacting with the NF-κB pathway (Jin et al., 2022). This pathway regulates inflammation and immune response, both of which are critical for successful bone repair. CDs help reduce inflammation by suppressing excessive NF-κB activity while promoting stem cell-mediated repair mechanisms. Furthermore, CDs influence macrophage polarization, facilitating the transition from pro-inflammatory (M1) to anti-inflammatory (M2) macrophages (Wan et al., 2022; Xu et al., 2024). This shift creates a favorable environment for tissue healing, minimizing inflammation-induced damage and improving overall bone regeneration.

## 5 CDs-based drug delivery systems

CDs have exceptional versatility in drug delivery due to their high drug-loading capacity and tunable surface chemistry. This section explores the methods and mechanisms by which CDs encapsulate or conjugate drugs, as well as the synergistic integration of drug-loaded CDs with other biomaterials to enhance therapeutic outcomes.

### 5.1 Physical encapsulation of drugs in CDs

Physical encapsulation refers to the non-covalent incorporation of drugs within the structural framework of CDs, often through post-synthetic processes. Non-covalent interactions, including hydrogen bonding, van der Waals forces, and hydrophobic interactions, facilitate drug retention within the CD framework. This method is advantageous due to its simplicity and adaptability, allowing researchers to modify drug-loading capacity according to specific therapeutic needs. One study entrapped riluzole within CDs fabricated from 2-acrylamido-2-methylpropanesulfonic acid (Algarra et al., 2024). An analysis of the interaction between CDs and riluzole revealed that van der Waals bonding (noncovalent interactions) is the sole mechanism of association. This results in the formation of drug-CD complexes without altering the molecular geometry of riluzole, which is crucial for preserving its bioactivity upon release from the CDs.

Melatonin CDs were encapsulated within a pH-sensitive hydrogel to enable stimuli-responsive release (Xie et al., 2025). It was observed that a sustained release of CDs occurred over 3 weeks, with a higher release rate in acidic conditions compared to neutral environments. The released melatonin CDs effectively scavenged intracellular reactive oxygen species (ROS) and demonstrated strong antibacterial activity.

**FIGURE 4:**
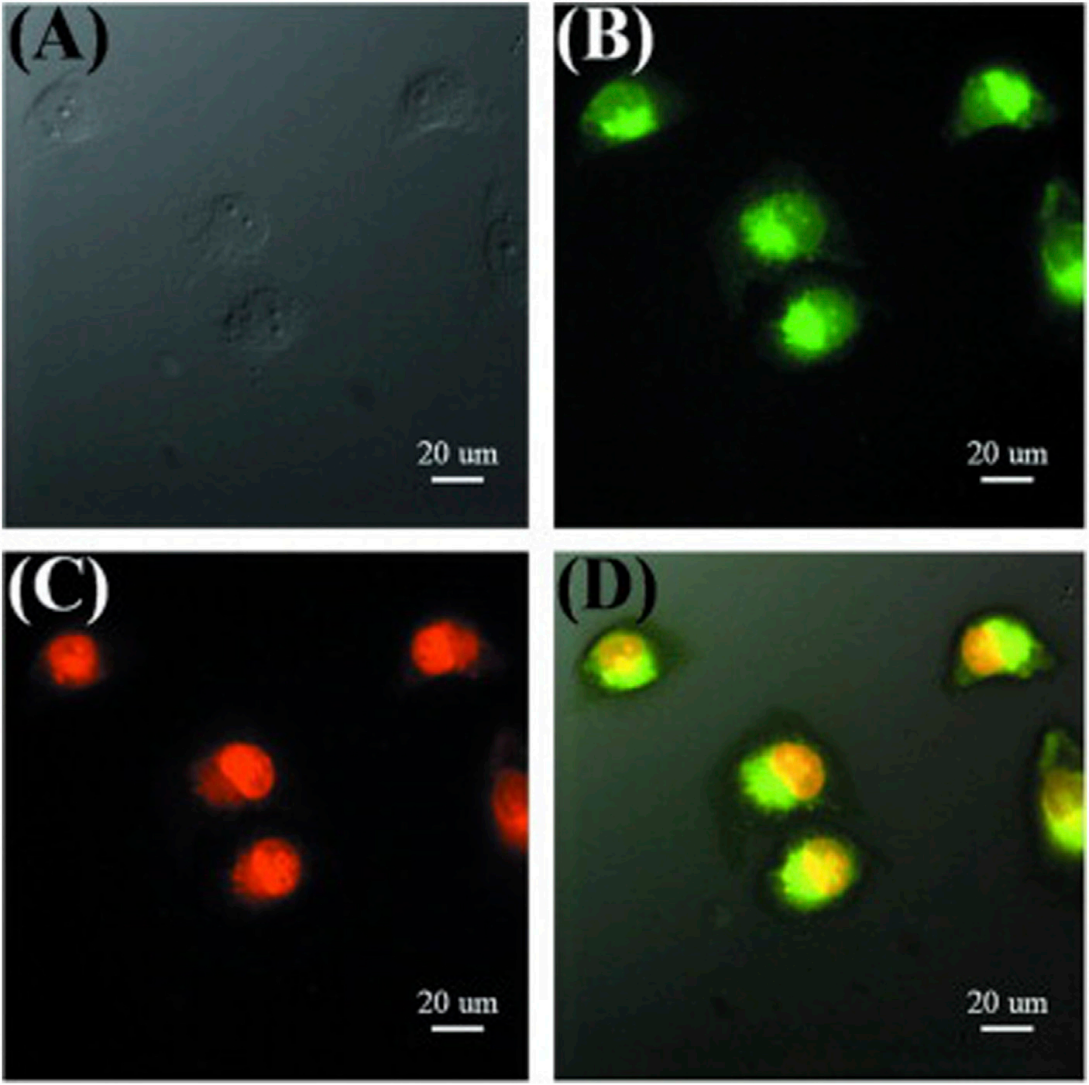
Fluorescence images of cells incubated with DOX-loaded CDs for 24 h **(A)** bright field, **(B)** excitation at 475 nm, **(C)** observed with the Cy 3 channel, **(D)** merged images. Adapted from Ref. (Wang et al., 2013), copyright 2013 Elsevier Ltd.

In another study, hollow CDs were synthesized from bovine serum albumin using a solvothermal method (Wang et al., 2013). The drug doxorubicin (DOX) was loaded into the core of the CDs by simply adding it to an aqueous solution of CDs and stirring the mixture overnight. This drug delivery system enables the detection of cellular internalization and the drug release process within cells. After incubation, green-colored CDs were observed to distribute throughout the cytoplasm and around the nuclei. Meanwhile, the bright red fluorescence of DOX was detected within the cell nuclei, indicating that the drug was almost completely released from the CDs and successfully entered the nuclei after 24 h (Figure 4; Wang et al., 2013).

Besides delivering conventional drugs, CDs can also be used to deliver genes using the physical encapsulation approach. For example, alendronate-polyethyleneimine-based CDs were developed as a gene vector for delivering the BMP-2 gene (Zhang et al., 2023). These CDs specifically target bone tissue while inhibiting osteoclast activity, allowing the delivery system to play a dual role in bone regeneration. By directly suppressing bone resorption and indirectly inducing bone formation through the transfection of osteogenic therapeutic genes, this approach holds a potential for enhancing bone repair.

Physical encapsulation presents several challenges, such as drug leakage and inconsistencies in loading efficiency. Addressing these issues requires further optimization to enhance the reliability of CD-based drug delivery systems.

### 5.2 Chemical conjugation of drugs onto CDs

Chemical conjugation is a highly effective strategy for drug delivery through CDs, involving the covalent attachment of therapeutic agents to the CD surface. This method enhances the stability of the drug-delivery system and allows for targeted delivery by incorporating receptor-binding ligands or molecular recognition elements. Unlike physical encapsulation, which relies on non-covalent interactions, chemical conjugation establishes strong molecular bonds between CDs and drug molecules, ensuring prolonged retention and controlled release. The ability to chemically modify CDs with various functional groups makes them an adaptable platform for precision medicine, offering improved therapeutic efficacy and reduced systemic toxicity.

Direct covalent bonding is a straightforward approach where reactive functional groups (e.g., carboxyl, hydroxyl, and amine groups) on CDs form stable bonds with corresponding functional groups on drug molecules. For example, carboxyl groups on glucose-based CDs formed amide bonds with amine group of BMP-2 via 1-ethyl-3-[3-dimethylaminopropyl]carbodiimide N-hydroxysuccinimide (EDC/NHS) mediated reaction, ensuring strong attachment without compromising drug activity (Rajabnejadkeleshteri et al., 2021). The BMP-2-CDs exhibited a high controlled delivery capacity and was sustainably released from the composite scaffold for up to 21 days. The bioactivity test showed the released BMP-2-CDs retained high bioactivity and promoted osteogenesis (Rajabnejadkeleshteri et al., 2021). In another report, the CDs synthesized from a bio-based raw material were chemically conjugated with four different peptides using the EDC/NHS method (Gogoi et al., 2017). Biofunctionalization with the peptides enhanced both the biocompatibility and osteoconductivity of the CDs (Gogoi et al., 2017). Similarly, zwitterionic CDs were surface-conjugated with collagen II peptides via the NHS/EDC coupling method, forming bifunctional CDs (Maity et al., 2023). These nucleus-targeting zwitterionic CDs function both as peptide delivery vehicles and as nano-agents for cartilage healing.

**FIGURE 5:**
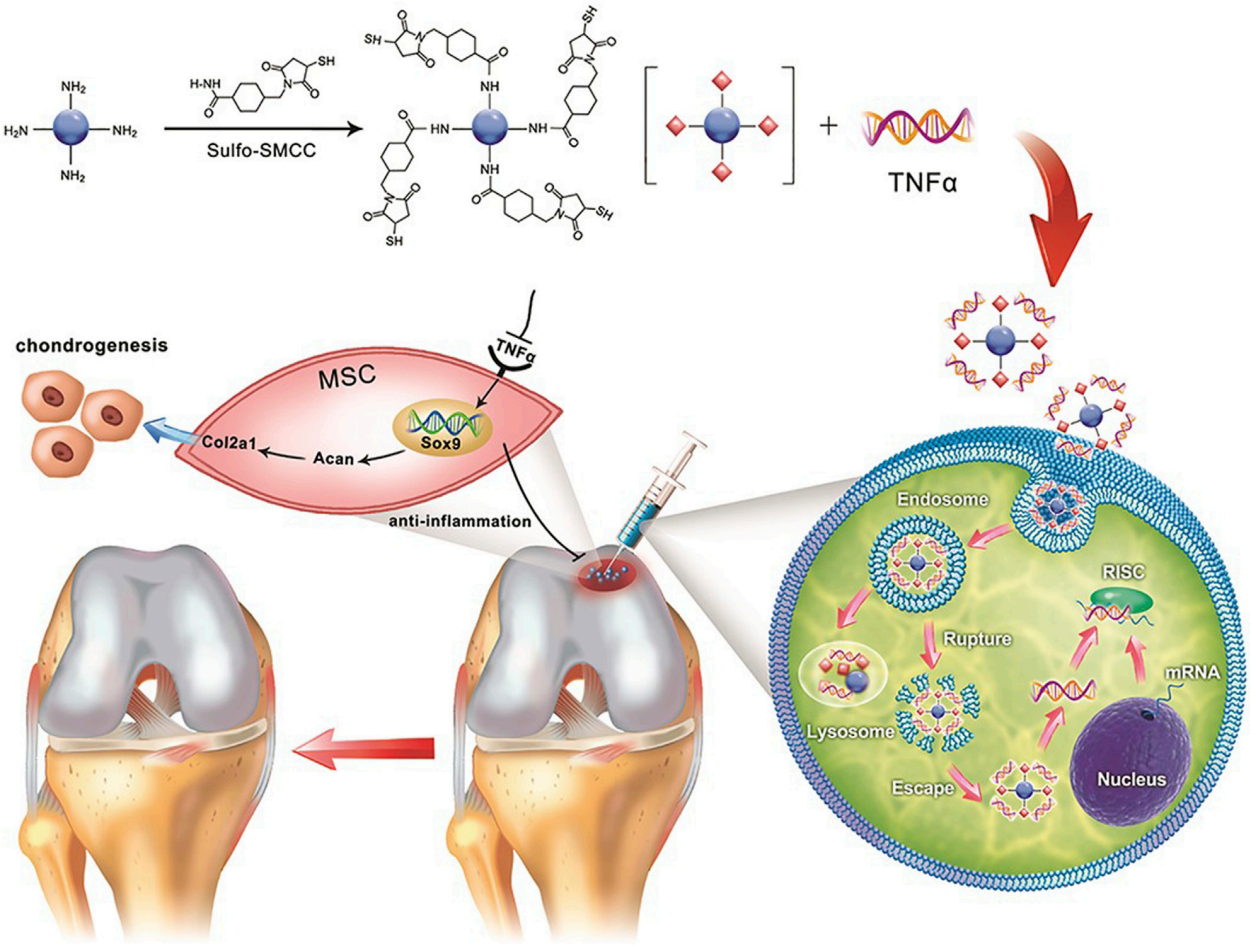
Schematic illustration depicting the use of the crosslinker sulfo-SMCC to form CD-SMCC-siTnfα complexes, enabling the development of a CD-based nanocarrier for gene delivery, real-time monitoring of cellular trafficking *in vitro*, and *in vivo* applications for tissue repair. Adapted from Ref. (Liu J. W. et al., 2019). Copyright ^©^ 2019 The Authors. Published by Wiley Periodicals, Inc. on behalf of AlphaMed Press.

Another chemical conjugation mechanism involves the use of cross-linking agents, such as sulfosuccinimidyl-4-(N-maleimidomethyl) cyclohexane-1-carboxylate (sulfo-SMCC), to enable efficient conjugation. These cross-linkers ensure stable drug attachment, preserving the therapeutic activity of both CDs and drugs. For example, CDs were modified with sulfo-SMCC to conjugate with si*Tnfα*, a small interfering RNA (siRNA) that silences the inflammatory cytokine tumor necrosis factor α (TNFα) (Figure 5). The conjugated CDs formed a stable thioether bond and retained the integrity of the siRNA (Liu J. W. et al., 2019).

Beyond controlled drug release, chemical conjugation offers enhanced stability, targeting precision, and multifunctionality. However, its effectiveness relies on careful optimization to balance conjugation efficiency and drug bioavailability. Preserving drug activity post-conjugation is essential for the successful application of chemically conjugated drug-loaded CDs in tissue regeneration.

### 5.3 Combination of drug-loaded CDs with other biomaterials

**FIGURE 6:**
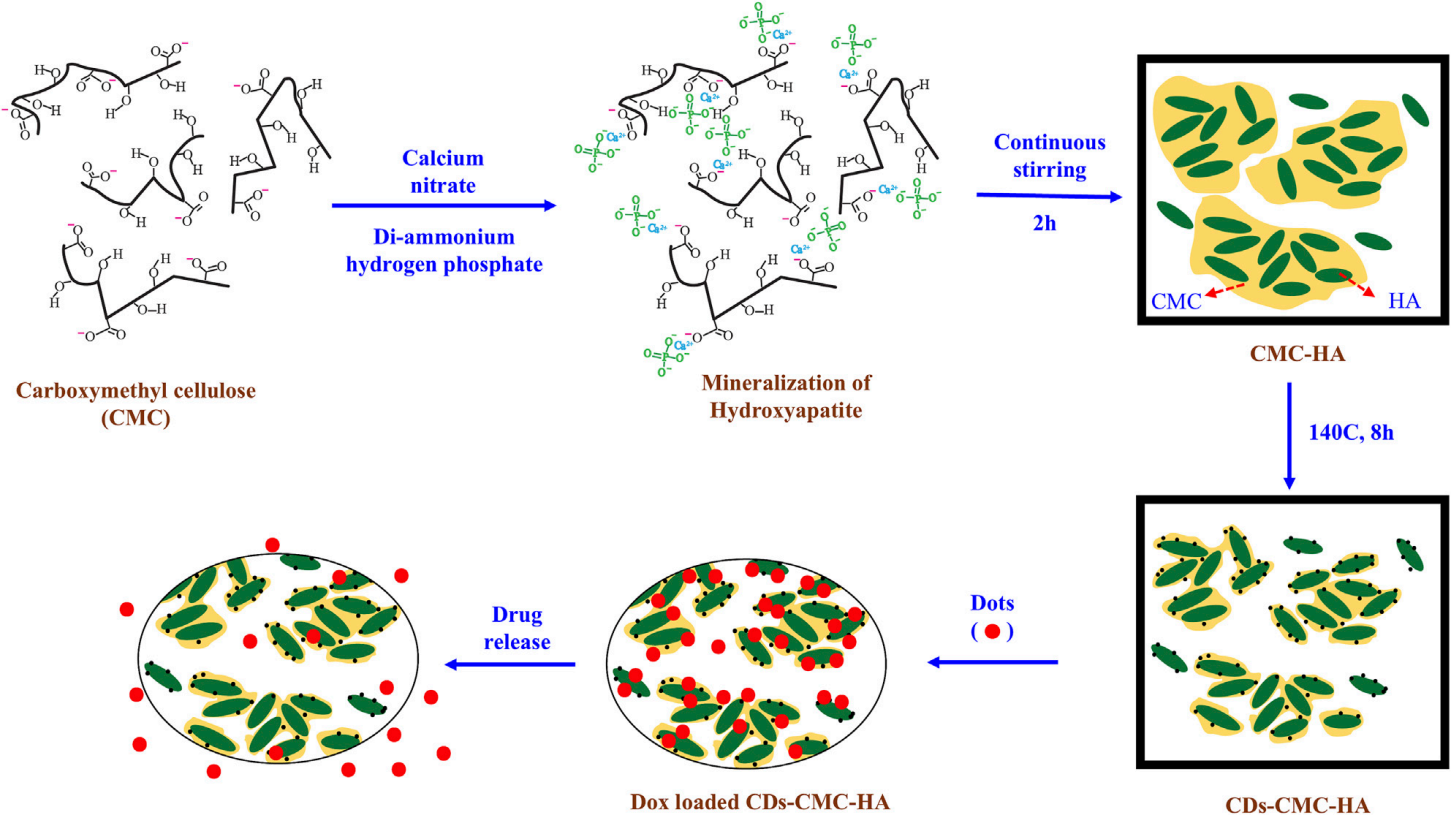
Schematic illustration of synthesizing CDs-decorated carboxymethyl cellulose-hydroxyapatite nanocomposite (CDs-CMC-HA) for doxorubicin delivery. Adapted from Ref. (Sarkar et al., 2018). Copyright ^©^ 2017 Elsevier Ltd.

CDs can be combined with ceramic-based systems such as hydroxyapatite (HA) to promote bone formation. In one study, CDs conjugated carboxymethyl cellulose-hydroxyapatite (CMC-HA) nanocomposites were synthesized by a simple one pot method (Sarkar et al., 2018). HA promoted the deposition of minerals that aid in bone regeneration, and when paired with CDs, it allowed for the efficient delivery of therapeutic mineral ions and growth factors. This combination enhanced the ability of CDs to stimulate osteogenesis. In addition, the CDs further loaded a chemotherapeutic drug to perform their dual functionalities (Figure 6). The *in vivo* release study showed the drug was constantly released from the CDs-CMC-HA for several days. The drug loading and release profile indicate that the drug molecules were bound with CDs-CMC-HA nanocomposite through electrostatic interaction with carboxyl group and released by a diffusion-controlled mechanism (Sarkar et al., 2018).

Metal-based systems also provide an exciting avenue for hybrid CD applications. Magnetic nanoparticles, when paired with CDs, allow for the development of magnetic field-guided drug delivery systems that improve targeting efficiency. This approach is beneficial for localized drug release, where external magnetic fields can be applied to direct CDs to specific tissues. In one study, superparamagnetic iron oxide nanoparticles (SPIONs) were doped into CDs to create FeCDs using a microwave-assisted single-step process (Das et al., 2019). The FeCDs were then encapsulated within a 3D-printed gelatin scaffold. When BMSCs were cultured on the scaffold, magnetic actuation of the FeCDs was employed to enhance their osteogenic differentiation.

The incorporation of biocompatible materials into CD-based delivery systems minimizes cytotoxicity and improves their interaction with biological environments, making them safer for long-term applications.

## 6 Applications of CDs-Based drug delivery for bone regeneration

CDs have emerged as a transformative tool in bone regeneration, leveraging their unique capabilities for targeted drug delivery, real-time imaging, and the regulation of biological processes. Their exceptional properties, including biocompatibility, photoluminescence, and surface functionalization, enable them to address various challenges in bone repair and regeneration. This section explores the key applications of CDs in bone regeneration, focusing on their role in bioimaging of BMSCs, regulation of the immune microenvironment in bone defects, and promotion of bone tissue regeneration.

### 6.1 Bioimaging

One of the most notable features of CDs is their excellent fluorescence properties. Unlike conventional quantum dots, which often contain toxic heavy metals such as cadmium, CDs are composed of carbon-based materials that exhibit minimal cytotoxicity. This makes them a safer alternative for biomedical applications, ensuring that stem cells remain viable and functional during the imaging process. Additionally, CDs display strong resistance to photobleaching, allowing researchers to conduct long-term tracking studies without significant signal loss. Their efficient cellular uptake further increases their utility in bioimaging, as BMSCs readily internalize CDs without disrupting normal physiological processes. This seamless integration ensures that labeled stem cells retain their regenerative potential while being effectively monitored.

**FIGURE 7:**
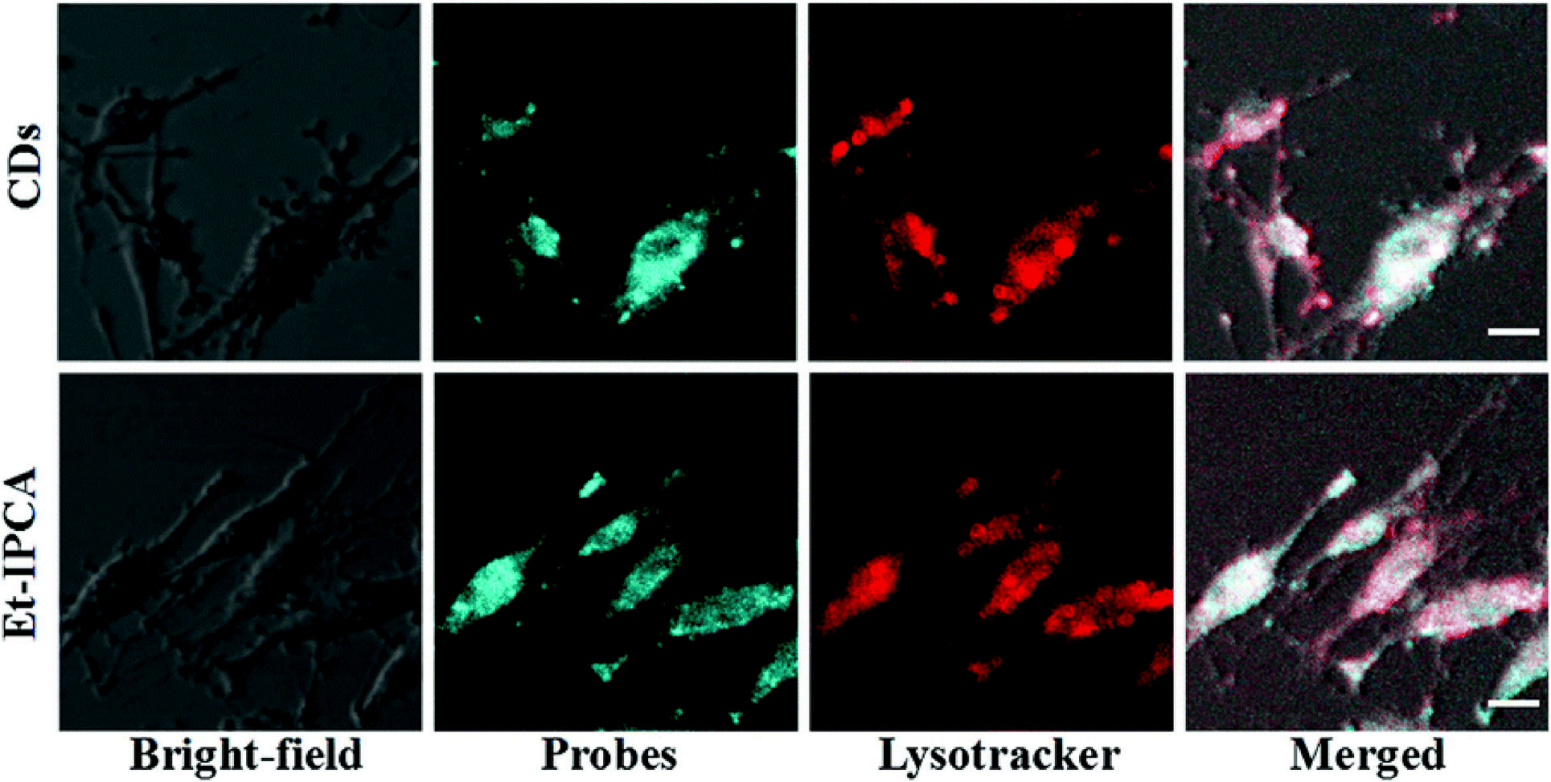
Confocal images of MSCs after incubated with CDs and Et-IPCA for 3 h. Scale bars: 10 μm. Adapted from Ref. (Shao et al., 2017). Copyright ^©^ 2017 Royal Society of Chemistry.

In one study, citric acid-based CDs and their derivatives, Et-IPCA, were fabricated for labeling and tracking BMSCs (Shao et al., 2017). After a 3-h incubation with CDs or Et-IPCA, BMSCs exhibited strong intracellular fluorescence. Overlay images revealed colocalization of fluorescent probes with LysoTracker-Red in cells, indicating that some nanoparticles entered endosomes or lysosomes, while others remained in the cytoplasm (Figure 7). Furthermore, extending the incubation period to 7 days resulted in consistent fluorescence signals in CD-treated BMSCs throughout the culture period. In contrast, Et-IPCA-treated cells displayed dimmer fluorescence signals, suggesting that CDs can serve as long-term labels for BMSCs (Shao et al., 2017).

In addition to labeling cells, CDs have also been utilized for bone tissue labeling. For instance, CDs synthesized from black carbon powder have exhibited a remarkable ability to bind specifically to calcified bones *in vivo* (Peng et al., 2017). This bone-specific binding capability is likely attributed to the distinctive surface chemistry of the CDs. This property highlights the potential of carbon nanopowder-derived CDs as bone-specific bioimaging agents and drug carriers.

CDs possess tunable photoluminescence properties, allowing for imaging across different wavelengths, including the visible and near-infrared (NIR) spectrum (Li et al., 2018). Their ability to emit fluorescence at longer wavelengths enhances deep tissue imaging, making them ideal candidates for stem cell tracking in complex biological environments.

One of the exciting aspects of CDs in bioimaging is their dual functionality in drug delivery. Since CDs can be engineered to carry therapeutic agents, researchers can simultaneously track BMSC behavior while delivering osteogenic or anti-inflammatory compounds to enhance bone regeneration (Liu J. W. et al., 2019). This integrated approach improves treatment precision, ensuring that stem cells receive targeted biochemical cues to optimize their differentiation and function. By using CDs as both imaging markers and drug carriers, regenerative therapies can be tailored to promote accelerated bone healing.

### 6.2 Regulation of immune microenvironment in bone defects

The immune microenvironment plays an essential role in bone regeneration, influencing the recruitment, proliferation, and differentiation of stem cells, osteoblasts, and other reparative cells. A well-regulated immune response ensures effective tissue healing by balancing inflammatory signals and promoting an environment conducive to osteogenesis. However, excessive inflammation or immune dysfunction can severely hinder bone regeneration, leading to chronic defects or impaired healing. CD-based interventions regulate this process by promoting the shift from a pro-inflammatory to an anti-inflammatory environment, thereby creating a conducive environment for bone tissue regeneration. Dracocephalum Moldavica L (DML), a traditional Chinese medicine rich in flavone compounds, exhibits strong antioxidant and anti-inflammatory properties. DML-derived CDs were synthesized using a one-pot hydrothermal method and demonstrated immunomodulatory effects on macrophage polarization (Zhang et al., 2024). Mechanistic studies indicate that these CDs promoted M2 macrophage polarization by rebalancing glycolysis and mitochondrial respiration in macrophages.

**FIGURE 8:**
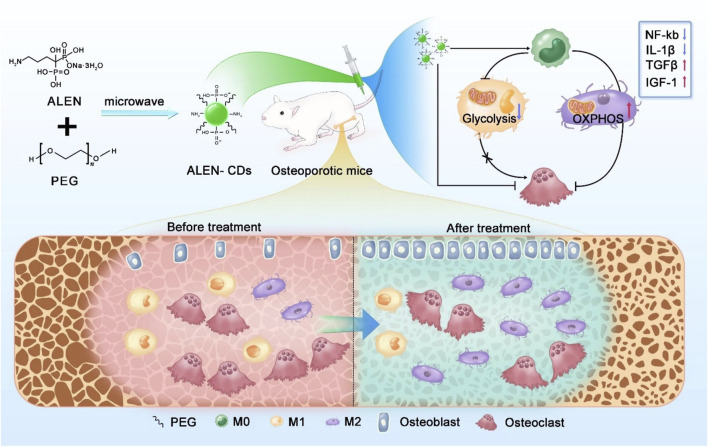
Schematic illustration of the synthesis of alendronate-derived CDs (ALEN-CDs) and their treatment for osteoporosis. Adapted from Ref. (Xu et al., 2024). Copyright ^©^ 2024 Elsevier Ltd.

In another study, alendronate-derived CDs were developed as bone-targeting agents to prevent bone resorption (Xu et al., 2024). Additionally, these CDs exhibited anti-inflammatory properties by regulating macrophage polarization from the pro-inflammatory M1 phenotype to the anti-inflammatory M2 phenotype (Figure 8). Animal experiments demonstrated that alendronate-derived CDs effectively reduced bone loss and improved osteoporosis in ovariectomized mice.

One of the primary advantages of CDs in bone regeneration is their intrinsic antioxidant and anti-inflammatory properties. Oxidative stress, often caused by excessive inflammation, can damage osteoblasts and stem cells, impairing their ability to form new bone tissue. CDs help mitigate this issue by scavenging ROS, thereby protecting cells from oxidative damage. For example, metformin-derived CDs exhibited strong ROS scavenging activity, effectively inhibiting synovium inflammation and fibrosis in collagen-induced arthritis rats (Zhang et al., 2025). Ultimately, these CDs prevented cartilage destruction and bone erosion. A detailed mechanistic analysis revealed that ROS elimination occurred through enzyme-like catalytic activity and suppression of the NOD-like receptor family, pyrin domain-containing 3 (NLRP3) inflammasome signaling pathway.

**FIGURE 9:**
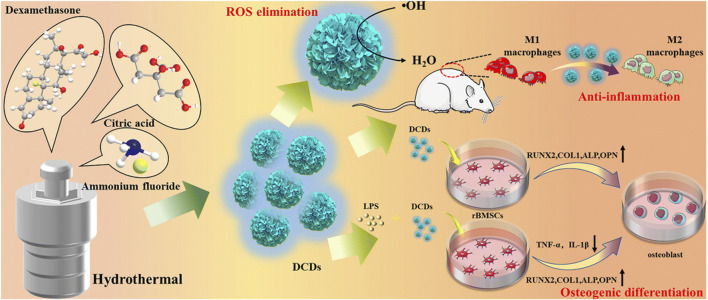
Schematic illustration of synthesizing dexamethasone-derived CDs and their anti-inflammatory and osteogenic differentiation activities. Adapted from Ref. (Wan et al., 2022). Copyright ^©^ 2022 Royal Society of Chemistry.

Dexamethasone-derived CDs also effectively scavenged ROS and reduced the inflammatory response (Wan et al., 2022). Additionally, they exhibited strong osteo-immunomodulatory activity, promoting the transition of macrophages from pro-inflammatory M1-type to anti-inflammatory M2-type under inflammatory conditions, ultimately accelerating bone regeneration (Figure 9). By modulating the immune microenvironment, CDs could help address one of the key barriers to effective bone regeneration, paving the way for more targeted and efficient therapies.

### 6.3 Promotion of bone tissue regeneration

CDs have emerged as promising nanomaterials for bone regeneration due to their multifaceted capabilities, including their ability to enhance osteogenic differentiation, promote extracellular matrix mineralization, support angiogenesis, and act as targeted drug delivery vehicles (Liu et al., 2020). Both *in vitro* and *in vivo* studies demonstrate their significant role in accelerating bone healing.

*In vitro* studies provide extensive insights into how CDs interact with osteoblasts, BMSCs, and extracellular matrix components to enhance bone formation. BMSCs are crucial precursor cells that give rise to osteoblasts, the primary bone-building cells. The presence of CDs enhances stem cell attachment, proliferation, and differentiation by mimicking the ECM, thereby facilitating cellular interactions. Moreover, CDs can be functionalized with osteogenic ions such as calcium and phosphate or with growth factors that accelerate BMSC differentiation into osteoblasts. For example, nitrogen-doped CDs were conjugated with HA nanoparticles using a hydrothermal co-precipitation technique (Khajuria et al., 2018). MC3T3-E1 osteoblast cells treated with those HA-conjugated CDs exhibited higher expression of osteogenic markers, such as ALP and RUNX2, and bone-related proteins such as OCN and OPN. The ability of CDs to upregulate these osteogenic markers highlights their potential for stimulating bone tissue regeneration.

Beyond enhancing BMSC differentiation, CDs play a crucial role in matrix mineralization, which is vital for forming structurally robust bone tissue. This process involves the deposition of HA, the mineral component of bone, within the ECM. CDs doped with calcium and phosphorus ions supply essential elements, facilitating calcium and phosphate deposition and accelerating mineralization in BMSCs (Wu et al., 2022).

*In vivo* studies provide compelling evidence of the effectiveness of CDs in accelerating bone healing. Research conducted on critical-sized bone defects has demonstrated that defects treated with CDs exhibited markedly faster healing, increased bone density, and improved mechanical stability compared to untreated controls (Geng et al., 2021). Furthermore, CD-modified scaffolds displayed higher osteoblast activity, facilitating the deposition and mineralization of ECM components necessary for strong and functional bone regeneration (Khajuria et al., 2018).

**FIGURE 10:**
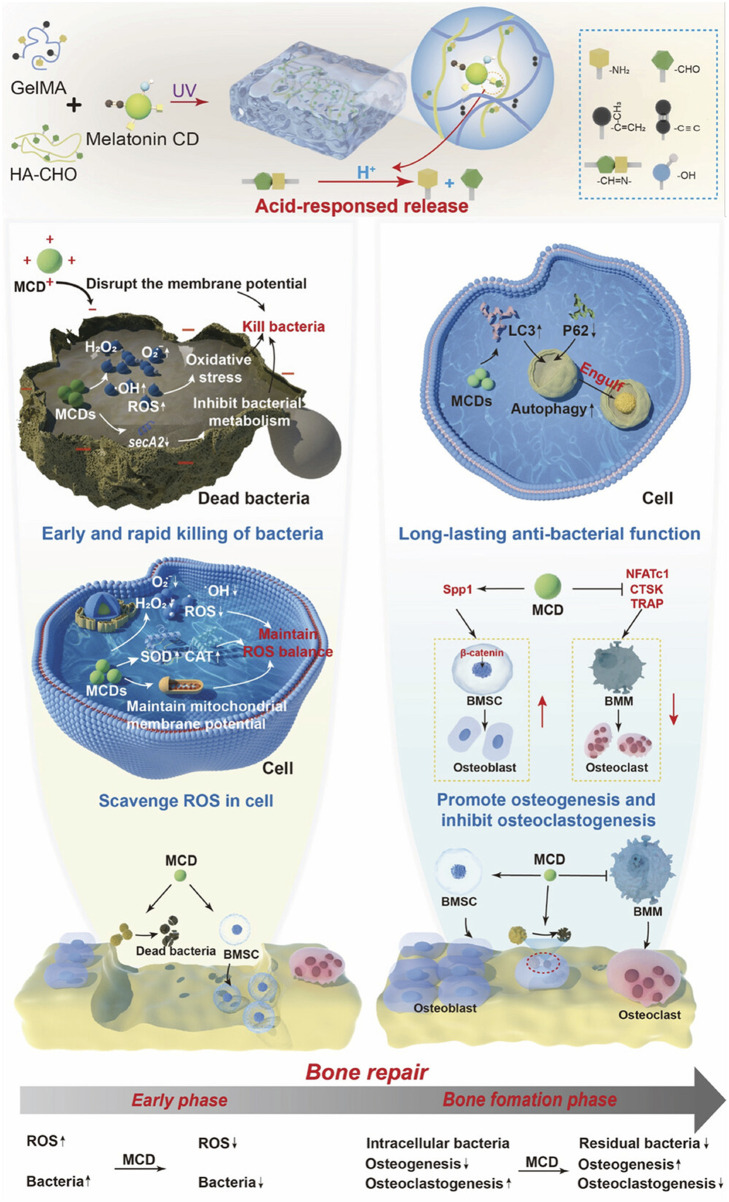
Melatonin-based CDs were encapsulated in a multifunctional hydrogel for enhanced repair of infectious bone defects. Adapted from Ref. (Xie et al., 2025). Copyright ^©^ 2025 The Author(s). Advanced Science published by Wiley‐VCH GmbH.

In addition to conventional pre-formed scaffold-based systems, CDs are being explored for injectable therapies, which offer minimally invasive solutions for targeted bone defect repair. CD-based injectable formulations allow localized treatment without requiring surgical intervention, reducing patient recovery time and minimizing procedural complications. This method is particularly useful for addressing areas that are difficult to access surgically, ensuring effective therapeutic outcomes with minimal discomfort. A CD-containing, pH-sensitive hydrogel was developed to enhance the repair of infectious bone defects (Xie et al., 2025). In this system, melatonin-derived CDs exhibited both antibacterial and osteogenic properties. During the early stage, the CDs eliminated bacteria and maintained intracellular ROS balance, creating a favorable microenvironment for osteoblast growth. Throughout the bone formation period, the CDs continued to eradicate intracellular bacteria while regulating the balance between osteogenesis and osteoclastogenesis to support bone repair (Figure 10). Meanwhile, the hydrogel responded to the acidic microenvironment by releasing CDs as needed. *In vivo* experiments revealed that after 4 weeks post-surgery, some new bone tissues were observed in the hydrogel group. In contrast, the CD-containing group exhibited complete coverage of the defect site, with new bone tissues integrating seamlessly with the surrounding bone. The bone volume fraction (BV/TV) in the CD-containing group reached approximately 25%, significantly surpassing that of the hydrogel and blank control groups. Moreover, by week eight, the bone surface in the CD-containing group was nearly fully repaired. Consistently, H&E staining images demonstrated enhanced bone formation in the CD-containing group. These results demonstrated that this CD-containing hydrogel system effectively facilitated the repair process of infectious bone defects in rats (Xie et al., 2025).

Another notable application of CDs in bone healing is their capacity to serve as controlled drug delivery vehicles. An ascorbic acid-PEI-based CDs were used as miR-2861 carriers for enhanced bone formation (Bu et al., 2020). miR-2861 is a micro-RNA targeting amino acid coding sequences of histone deacetylase 5 (HDAC5) that is involved in degradation of RUNX2, resulting in promoting bone formation. The CDs demonstrated excellent fluorescence stability and were effectively internalized into BMSCs, showing strong osteogenic effects *in vitro*. Remarkably, the CDs and miR-2861 acted synergistically to enhance osteogenic differentiation and promote new bone regeneration. In a calvarial bone defect model, the miRNA-loaded CDs group exhibited greater new bone formation, covering approximately 71% of the bone defect area 12 weeks post-operation—significantly surpassing the two other control groups. By combining drug delivery with bioimaging, CDs enable researchers to monitor the progress of healing while simultaneously enhancing bone repair (Bu et al., 2020).

**FIGURE 11:**
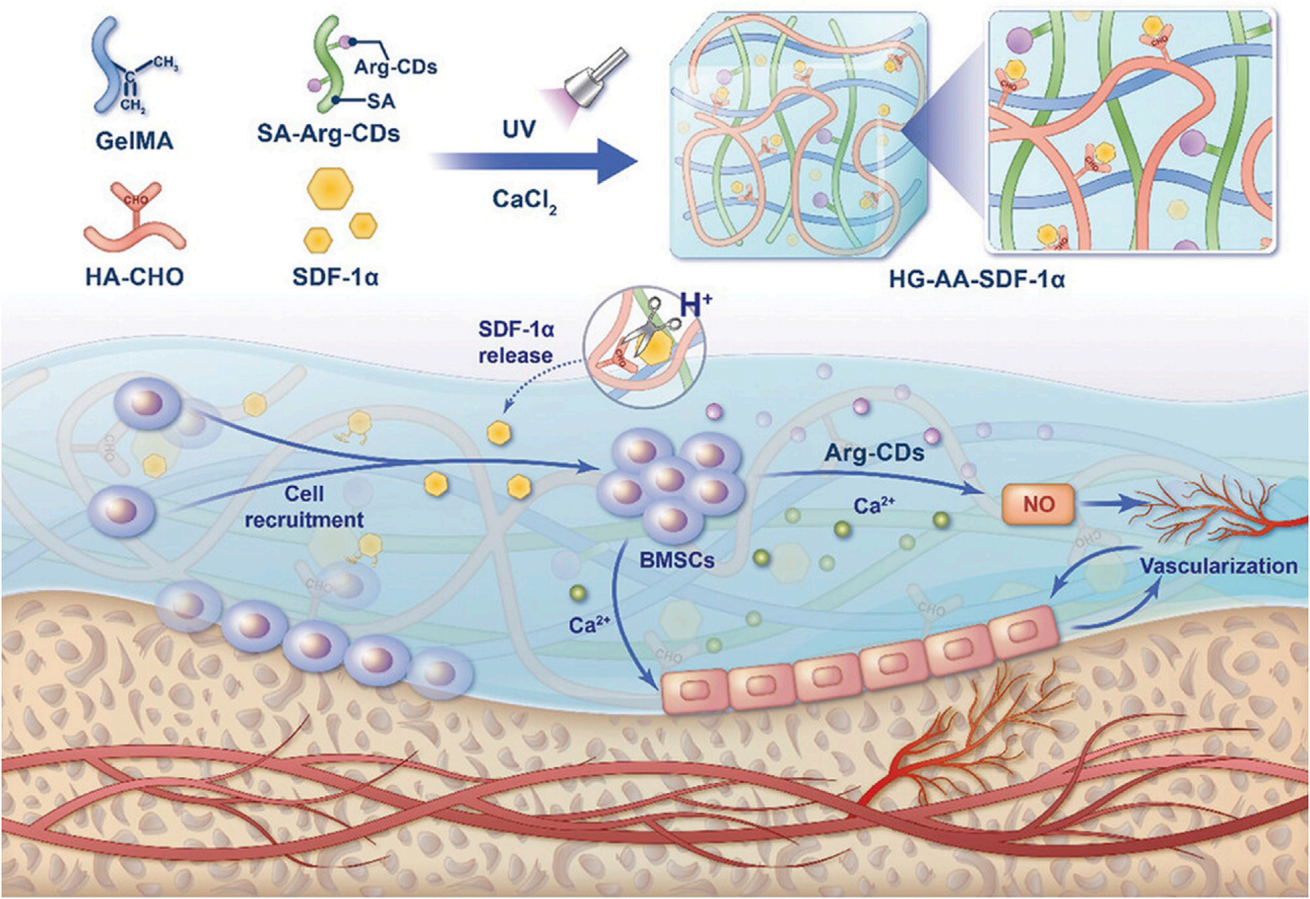
Schematic illustration of a composite hydrogel that couples osteogenesis and angiogenesis to promote bone regeneration. Adapted from Ref. (Xiao et al., 2025). ^©^ 2025 The Author(s). Advanced Science published by Wiley‐VCH GmbH.

Beyond direct osteogenesis, CDs significantly contribute to angiogenesis and vascularization, ensuring the adequate formation of blood vessels within the regenerating bone tissue. Nitric oxide (NO) is a bioactive substance that induces vasodilation during inflammation, and increases the blood flow to fracture healing tissue (Lundberg et al., 2015). Mechanistically, NO activates the NO-cyclic guanosine monophosphate (cGMP) signaling pathway and coordinates the angiogenic-osteogenic coupling to accelerate bone repair. In a recent publication, a composite hydrogel was developed with a dual-control switch function to recruit and stimulate osteogenic differentiation of BMSCs in bone damage areas (Figure 11) (Xiao et al., 2025) The hydrogel, formed by integrating Arg carbon dots (Arg-CDs) with various biomaterials, responded to the acidic microenvironment at bone injury sites by releasing stromal cell-derived factor-1α (SDF-1α) and rapidly recruiting BMSCs. The recruited BMSCs metabolized Arg-CDs to produce NO, promoting angiogenesis and creating an improved osteogenic environment. Additionally, free Ca2+ released from the hydrogel further enhanced BMSC differentiation. A cranial defect model was established, and μ-CT results revealed that the amount of new bone formation in the CDs-containing SDF-1α group was two to three times higher than in the other groups. This hydrogel effectively couples osteogenesis and angiogenesis to synergistically promote bone regeneration, offering a promising strategy for bone repair.

Overall, CDs provide numerous benefits, including promoting osteogenesis, supporting vascularization, modulating immune responses, and enabling targeted drug delivery. Their ability to accelerate bone healing *in vivo*, along with their integration into scaffold-based, injectable, and stem cell-based therapies, positions them as promising candidates for bone tissue regeneration.

## 7 Challenges and future perspectives

CDs offer a multifunctional approach to improving osteogenic differentiation, matrix mineralization, angiogenesis, and controlled drug delivery. Their biocompatibility, fluorescence capabilities, and regenerative properties make them valuable tools in advancing regenerative medicine. However, several challenges must be overcome to achieve successful clinical translation for bone tissue regeneration. One major concern is the cytotoxicity and long-term stability of CDs. While CDs are often praised for their biocompatibility, variations in synthesis methods, surface functionalization, and exposure levels can result in cytotoxic effects. Understanding their degradation pathways, long-term retention in biological systems, and possible toxicity at higher concentrations is essential for ensuring safe therapeutic use. Extensive *in vitro* and *in vivo* studies are required to determine their stability under physiological conditions and assess any potential adverse effects that might arise from prolonged exposure.

Another problem is the diverse fabrication methods used to synthesize CDs, which result in significant variations in their structural, chemical, and functional properties. These differences make direct comparisons between fabrication techniques and the properties of CDs difficult, if not impossible. To advance the field, future research should prioritize systematic comparative analyses of CD preparations, utilizing standardized chemical and biological assays. Establishing uniform evaluation criteria will enable researchers to better assess and optimize CDs for their practical utility.

Another challenge lies in advancing CD-based drug delivery systems (Zheng et al., 2025). Although CDs have demonstrated promising abilities for controlled drug release, there is a growing need for more sophisticated delivery mechanisms that respond to specific stimuli, ensure long-term sustained release, and enable the co-delivery of multiple therapeutic agents. By integrating CDs with stimuli-responsive systems—such as pH-sensitive, temperature-responsive, or enzyme-triggered release platforms—researchers can enhance their precision in delivering drugs to targeted bone defect sites. The ability to load and release different therapeutic compounds simultaneously would further optimize bone regeneration processes, improving healing outcomes. Meanwhile, enhancing the targeting properties of CDs through optimized structural design—such as size, shape, and surface charge—would be highly advantageous.

Cell tracking is another key hurdle in CD research. Traditional imaging methods often fall short in providing precise long-term cell tracking capabilities due to signal degradation or insufficient brightness. CDs, with their tunable optical properties, offer significant advantages for bioimaging applications, yet improvements are needed to achieve long-wavelength emission, multiple colors for multiplexed imaging, and ultra-bright fluorescence for enhanced detection sensitivity. These features are particularly important for monitoring bone regeneration processes and understanding cellular interactions within complex tissue environments. Developing CDs with optimized fluorescence characteristics will facilitate high-resolution tracking of stem cells and immune cells involved in bone repair, contributing to better therapeutic strategies.

The underlying mechanisms through which CDs regulate bone stem cells and immune cells remain incompletely understood. CDs have shown promise in modulating cellular behaviors, including promoting osteogenic differentiation and influencing immune cell responses. However, deeper insights into their molecular interactions and pathways are needed to fully harness their regenerative potential. Investigating how CDs interact with key signaling molecules, ECM components, and inflammatory mediators will allow researchers to fine-tune their properties for better regenerative efficacy. Addressing these questions is essential for optimizing CD-based therapies in bone tissue engineering. Moreover, when CDs are examined *in vivo*, expanding biological assays to include transcriptome analysis is essential for uncovering previously overlooked molecular mechanisms. Current studies primarily focus on well-established pathways, potentially missing key regulatory networks within CD-based biomaterial systems that influence bone regeneration. A comprehensive analysis of gene expression profiles could reveal novel signaling cascades and interactions that contribute to bone regeneration.

The transition of CD-based drug delivery systems from laboratory research to clinical applications for bone regeneration is currently hindered by several factors. First, research remains largely limited to *in vitro* and small-animal studies, necessitating extensive preclinical evaluations to assess long-term biosafety, biodistribution, and potential toxicity in human models. Additionally, regulatory hurdles, including approval from governing agencies, require comprehensive validation of CDs’ therapeutic efficacy and stability. Scalability presents another major challenge. While laboratory-scale production methods yield promising results, translating these processes into large-scale manufacturing with consistent quality is difficult. Variations in precursor materials, synthesis conditions, and purification steps can lead to inconsistencies in CD properties, affecting their performance in tissue regeneration. Standardizing synthesis protocols and developing scalable production techniques will be critical for making CDs commercially viable for clinical applications. Addressing these challenges through interdisciplinary collaboration will be crucial to bridging the gap between laboratory innovation and clinical applications at the bedside.
